# Retrospective analysis of the efficacy and survival associated with cTACE and DEB-TACE in the palliative treatment of hepatocellular carcinoma: experience of a tertiary care hospital in southern Brazil

**DOI:** 10.1590/0100-3984.2023.0105

**Published:** 2024-05-07

**Authors:** Priscila Cavedon Fontana, Gabriela Perdomo Coral, Alex Finger Horbe, Raquel de Freitas Jotz, Beatriz Garcia de Morais, Angelo Alves de Mattos

**Affiliations:** 1 Universidade Federal de Ciências da Saúde de Porto Alegre (UFCSPA), Porto Alegre, RS, Brazil; 2 Irmandade Santa Casa de Misericórdia de Porto Alegre (ISCMPA), Porto Alegre, RS, Brazil

**Keywords:** Carcinoma, hepatocellular, Chemoembolization, therapeutic, Microspheres, Survival analysis, Carcinoma hepatocelular, Quimioembolização terapêutica, Microesferas, Análise de sobrevida

## Abstract

**Objective:**

To compare conventional transarterial chemoembolization (cTACE) and drug-eluting bead TACE (DEB-TACE) in terms of efficacy, survival, and adverse effects in patients with hepatocellular carcinoma who are not candidates for curative therapy.

**Materials and Methods:**

This was a retrospective study of patients with hepatocellular carcinoma who underwent cTACE or DEB-TACE for palliative treatment between January 2009 and December 2021. The Kaplan-Meier method was used for survival analysis. Values of *p* < 0.05 were considered statistically significant.

**Results:**

We evaluated 268 patients, of whom 70 underwent DEB-TACE and 198 underwent cTACE. There was no significant difference between the groups regarding sex, age, or etiology of cirrhosis. The proportion of patients achieving a complete response on imaging examinations was higher in the cTACE group (31.8% vs. 16.1%), whereas that of patients achieving a partial response was higher in the DEB-TACE group (33.9% vs.19.7%), and the differences were significant (*p* = 0.014). The mortality rate was similar between the groups. The survival rate in the DEB-TACE and cTACE groups, respectively, was 87.0% and 87.9% at one year, 35.1% and 32.9% at three years, and 20.5% and 18.1% at five years (*p* = 0.661). There was no significant difference between the DEB-TACE and cTACE groups in terms of the frequency of adverse events (7.1% vs. 17.8%; *p* = 0.052). The most common complication in both groups was post-embolization syndrome.

**Conclusion:**

Although a complete response was more common among the patients who underwent cTACE, there was no difference in survival between the groups and the frequency of adverse events was similar.

## Abstract (pt)

**Objetivo:**

Comparar a eficácia, sobrevida e efeitos adversos entre cTACE e DEB-TACE em pacientes com carcinoma hepatocelular não candidatos a terapia curativa.

**Materiais e Métodos:**

Estudo retrospectivo de pacientes com carcinoma hepatocelular submetidos a cTACE ou DEB-TACE para tratamento paliativo entre janeiro de 2009 e dezembro de 2021. Foi utilizado o método Kaplan-Meier para análise de sobrevida. Valor de *p* < 0,05 foi considerado estatisticamente significante.

**Resultados:**

Foram avaliados 268 pacientes, dos quais 70 foram submetidos a DEB-TACE e 198 foram submetidos a cTACE. Não houve diferença em relação ao sexo, idade e etiologia da cirrose. O grupo cTACE apresentou maior porcentual de resposta completa em exames de imagem (31,8% vs. 16,1%) e o grupo DEB-TACE apresentou maior porcentual de resposta parcial (33,9% vs.19,7%), com valor de *p* = 0,014. A mortalidade foi semelhante. As taxas de sobrevivência para os grupos DEB-TACE e cTACE foram 87,0% e 87,9% em um ano, 35,1% e 32,9% em três anos e 20,5% e 18,1% em cinco anos, respectivamente (*p* = 0,661). Em relação à frequência de eventos adversos, não houve diferença significativa entre os grupos (7,1% na DEB-TACE vs. 17,8% na cTACE; *p* = 0,052). A complicação mais comum, em ambos os grupos, foi a síndrome pós-embolização.

**Conclusão:**

Embora tenha sido observada maior frequência de resposta completa em pacientes submetidos a cTACE, não houve diferença na sobrevida dos pacientes entre os grupos. A taxa de eventos adversos também foi semelhante.

## INTRODUCTION

Hepatocellular carcinoma (HCC) is the most common primary malignant neoplasm of the liver, accounting for 75% of all malignant liver tumors worldwide^(1)^. It is also the sixth most prevalent neoplasm and the fourth leading cause of cancer-related mortality. The prognosis is poor in all regions of the world and, in 2018, the overall incidence of liver neoplasia was 9.3 per 100,000 person-years and the associated mortality rate was 8.5 per 100,000 person-years, indicating a very close relationship between incidence and mortality^(2,3)^.

There are multiple risk factors for HCC, and one of the features common to many of them is the presence of cirrhosis^(4)^. Approximately one-third of patients with cirrhosis develop HCC during their lifetime^(5)^. The main risk factors for HCC are liver cirrhosis *per se*, infection with hepatitis B or C virus, alcoholism, metabolic dysfunction-associated steatotic liver disease, hemochromatosis, and ingestion of environmental toxins such as aflatoxin^(6)^.

It is estimated that only 10-30% of patients diagnosed with HCC are eligible for curative treatment^(7)^. For patients with liver tumors who are not eligible for resection, ablation, or transplantation, treatment options include palliative methods such as transarterial chemoembolization (TACE), drug-eluting bead TACE (DEB-TACE), transarterial radioembolization, and systemic therapy^(8)^.

The TACE method was introduced in 1977 by Yamada et al., who applied it in a cohort of 120 patients^(9)^. The conventional TACE (cTACE) technique involves intra-arterial injection of cytotoxic agents such as doxorubicin, cisplatin, epirubicin, mitomycin, and irinotecan, which are emulsified in the oil-based radiopaque contrast agent, lipiodol. That is followed by injection of embolic agents, resulting in embolization of the tumor microcirculation, which leads to ischemic necrosis. The lipiodol causes retention of the chemotherapeutic agents within the tumor and can be detected by imaging after the procedure, predicting the response to treatment. However, in cTACE, the tumor does not always retain lipiodol, resulting in decreased effectiveness of therapy and risk of liver damage^(10-12)^.

In 2010, DEB-TACE was introduced in order to reduce side effects and improve the overall results of TACE^(12)^. The DEB-TACE method uses non-absorbable embolic microspheres (beads) that elute cytotoxic drugs, allowing the drugs to be slowly released into the lesion. The use of microspheres also allows deeper distal embolization of small vessels, causing selective occlusion of the arteries that feed the tumor^(13,14)^. Studies comparing the efficacy of cTACE and DEB-TACE have produced controversial results, showing similar efficacy trends but a lower rate of adverse effects for DEB-TACE^(14-16)^.

This aim of this study was to compare cTACE and DEB-TACE in terms of survival and adverse events in patients undergoing the procedures for the palliative treatment of HCC.

## MATERIALS AND METHODS

This was a retrospective study conducted at the Irmandade Santa Casa de Misericórdia de Porto Alegre, a tertiary care hospital in the city of Porto Alegre, RS, Brazil. We reviewed the medical records of all consecutive patients ≥ 18 years of age who were diagnosed with HCC and underwent cTACE or DEB-TACE for palliative treatment between January 2009 and December 2021. The study was approved by the Research Ethics Committee of the Hospital (Reference no. 3473656). Patients who had undergone both cTACE and DEB-TACE were excluded, as were those who had undergone hepatectomy or other therapeutic modality prior to TACE, those for whom the medical records were incomplete, and those who underwent TACE as neoadjuvant therapy prior to liver transplantation.

The diagnosis of HCC was made according to the criteria established by the American Association for the Study of Liver Diseases^(17)^, using triphasic abdominal computed tomography (CT), magnetic resonance imaging with gadolinium, or both as the dynamic imaging methods. In cases in which diagnosis was not possible with imaging methods, liver biopsy was performed.

The following patient characteristics were evaluated: age; sex; etiology of cirrhosis; Child-Pugh class; and model for end-stage liver disease (MELD) score. Regarding HCC, the variables studied were as follows: diagnostic method; Barcelona Clinic Liver Center (BCLC) stage; alpha-fetoprotein (AFP) level; diameter of the largest neoplastic nodule; number of nodules; presence of portal vein thrombosis; and the location of nodules. Regarding the cTACE and DEB-TACE procedures, the following were evaluated: type of catheterization (selective or superselective); type of chemotherapy used; number of sessions; complications; and follow-up imaging. We also evaluated overall survival and the cause of death.

The response to TACE was described in accordance with the Modified Response Evaluation Criteria in Solid Tumor (mRECIST) criteria^(18)^. The mRECIST category was determined after re-evaluation by an independent radiologist, one to two months after the procedure. Patients were followed until death or until the end of the study period (December 2021).

Data were stored in an MS Excel spreadsheet and subsequently analyzed with the IBM SPSS Statistics software package, version 28.0 (IBM Corp., Armonk, NY, USA). Quantitative variables were expressed as mean and standard deviation or as median and interquartile range. Categorical variables were expressed as absolute frequency and percentage. The means were compared with Student’s t-test. For variables with asymmetric data distribution, the Mann-Whitney test was applied. In the comparison of proportions, the chi-square test or Fisher’s exact test was used. In the comparison between the AFP levels at diagnosis and those observed after cTACE or DEB-TACE, the Wilcoxon test was applied. Survival time was estimated by plotting Kaplan-Meier curves and was compared between groups by log-rank test. To adjust for confounding factors, multivariate models of Cox proportional hazards regression (for death), Poisson (for complications), and multinomial logistics (for the mRECIST category) were applied. Values of *p* < 0.05 were considered statistically significant.

## RESULTS

Between January 10, 2009 and December 31, 2021, a total of 328 patients with HCC underwent TACE for palliative treatment. A total of 60 patients were excluded: 18 because they had undergone both procedures (cTACE and DEB-TACE); 3 because they had also undergone radiofrequency ablation; 11 because they had undergone hepatectomy prior to TACE; and 28 because they did not undergo follow-up examinations. Therefore, the final sample comprised 268 patients, of whom 70 had undergone DEB-TACE and 198 had undergone cTACE. Patients characteristics are shown in Table 1.

**Table 1:** Sociodemographic and clinical characteristics of patients with HCC undergoing TACE.

Variable	Type of TACE		*P*
	DEB-TACE (n = 70)	cTACE (n = 198)	
Age (years), mean ± SD	65.3 ± 12.3	66.8 ± 10.1	0.333
Sex, n (%)			0.237
Male	53 (75.7)	133 (67.2)	
Female	17 (24.3)	65 (32.8)	
Cirrhosis, n (%)			0.040
No	6 (8.7)	5 (2.6)	
Yes	63 (91.3)	189 (97.4)	
Etiology, n (%)			0.130
Hepatitis C	38 (60.3)	107 (56.6)	
Alcohol use	8 (12.7)	28 (14.8)	
Hepatitis B	2 (3.2)	8 (4.2)	
Nonalcoholic fatty liver disease	5 (7.9)	7 (3.7)	
Hepatitis C + alcohol use	3 (4.8)	29 (15.3)	
Hepatitis B + alcohol use	1(1-6)	1 (0.5)	
Hepatitis B + hepatitis C	0 (0.0)	2 (1.1)	
Cryptogenic	3 (4.8)	6 (3.2)	
Hemochromatosis	1(1-6)	0 (0.0)	
Other	2 (3.2)	1 (0.5)	
Child-Pugh class, n (%)			0.449
A	51 (85.0)	143 (79.4)	
B	9 (15.0)	37 (20.6)	
MELD score, mean ± SD	10.8 ± 5.3	11.2 ± 4.9	0.601
BCLC stage, n (%)			0.757
A	15 (21.4)	35 (17.8)	
B	48 (68.6)	144 (73.1)	
C	7 (10.0)	18 (9.1)	

There was no significant difference between the groups regarding sex or age: in the DEB-TACE group, 75.7% of the patients were men and the mean age was 65.3 years; in the cTACE group, 67.3% were men and the mean age was 66.8 years. However, there was a significant difference between the groups regarding the presence of cirrhosis, which was identified in 91.3% of the patients in the DEB-TACE group and in 97.4% of those in the cTACE group. In both groups, the most common etiologies of cirrhosis were infection with hepatitis C virus and excessive alcohol use. Most of the patients (85.0% and 79.4% in the DEB-TACE and cTACE groups, respectively) were categorized as Child-Pugh class A, and the MELD score did not differ significantly between the two groups (10.8 and 11.2, respectively). Most of the tumors were classified as BCLC stage B.

Table 2 shows aspects related to the tumor, the therapeutic technique employed, and the evolution of the patients. In both groups, the diagnosis of HCC was predominantly made by imaging methods (in 95.7% and 94.4% in the DEB-TACE and cTACE groups, respectively). The majority of the neoplastic lesions were located in the left hepatic lobe, that region being targeted by TACE in 54.3% of the patients in the DEB-TACE group and in 64.6% of those in the cTACE group (*p* = 0.190). In both groups, the median number of nodules was two and portal vein thrombosis was present in less than 10% of all cases.

**Table 2:** Comparison between DEB-TACE and cTACE in terms of the characteristics of the patients and their tumors.

Variable	Type of TACE		*P*
	DEB-TACE (n = 70)	cTACE (n = 198)	
Method(s) used for the diagnosis of HCC, n (%)			0.915
Imaging	67 (95.7)	187 (9.4)	
Biopsy	1 (1.4)	4 (2.0)	
Imaging + biopsy	2 (2.9)	7 (3.5)	
Number of nodules, median (interquartile range)	2 (1-2.5)	2 (1-2.5)	0.719
Diameter of the largest nodule (cm), median (interquartile range)	3.95 (3.1-5.8)	4.3 (2.9-6.1)	0.908
Target segment, n (%)			0.190
Right lobe	17 (24.3)	44 (22.2)	
Left lobe	38 (54.3)	128 (64.6)	
Right lobe + left lobe	15 (21.4)	26 (13.1)	
Imaging characteristic, n (%)			0.082
Typical (LI-RADS 4 or 5)	66 (94.3)	192 (98.5)	
Atypical (LI-RADS 1,2, or 3)	4 (5.7)	3 (1.5)	
Portal thrombosis, n (%)			0.488
None	55 (90.2)	172 (94.5)	
Tumor-related	5 (8.2)	8 (4.4)	
Non-tumor-related	1 (1.6)	2 (1.1)	
Adverse event, n (%)			0.052
No	65 (92.9)	162 (82.2)	
Yes	5 (7.1)	35 (17.8)	
Type of adverse event, n (%)			0.837
Post-embolization syndrome	4 (80.0)	25 (71.4)	
Vascular	0 (0.0)	4 (11.4)	
Infectious	0 (0.0)	1 (2.9)	
Other	1 (20.0)	5 (14.3)	
Successful catheterization, n (%)			0.653
No	2 (2.9)	4 (2.0)	
Yes	68 (97.1)	194 (98.0)	
Type of catheterization, n (%)			0.655
Superselective	56 (86.2)	166 (89.2)	
Selective	9 (13.8)	20 (10.8)	
AFP level (ng/dL) at diagnosis, median (interquartile range)	16.9 (5.6-101.0)	30.7 (7.7-248.0)	0.192
Number of TACE procedures, median (interquartile range)	2(1-2)	1(1-2)	0.128
mRECIST response of the target lesion, n (%)			0.014
Complete	9 (16.1)	55 (31.8)	
Partial	19 (33.9)	34 (19.7)	
Stable disease	1 (1.8)	14 (8.1)	
Progressive disease	27 (48.2)	70 (40.5)	
AFP level (ng/dL) after TACE, median (interquartile range)	15.5 (5.1-265.0)	31.7 (6.1-388.0)	0.494
Death, n (%)			0.141
No	24 (34.3)	48 (24.2)	
Yes	46 (65.7)	150 (75.8)	
Cause of death, n (%)			0.946
Unrelated to the tumor	7 (31.8)	27 (35.5)	
Related to the tumor	15 (68.2)	49 (64.5)	

Nearly all of the patients underwent successful catheterization, which was of the superselective type in more than 80%. In the sample as a whole, the chemotherapy used was doxorubicin and a median of two chemoembolization procedures were performed. When evaluating the response after treatment of the target lesion, we found that the proportion of patients achieving a complete response was higher in the cTACE group (31.8% vs. 16.1%), whereas that of patients achieving a partial response was higher in the DEB-TACE group (33.9% vs.19.7%), and the differences were significant (*p* = 0.014). The median AFP level at diagnosis was 16.9 ng/dL and 30.7 ng/dL in the DEB-TACE and cTACE groups, respectively (*p* = 0.192), whereas it was 15.5 ng/dL and 31.7 ng/dL, respectively, after TACE (*p* = 0.494).

**Figure 1:**
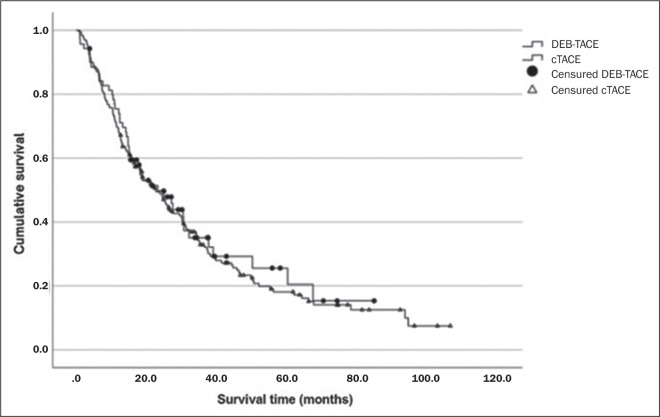
Kaplan-Meier survival curve comparing patients treated with cTACE and those treated with DEB-TACE (p = 0.661).

Of the 70 patients in the DEB-TACE group, 46 (65.7%) died during the study period, compared with 150 (75.8%) of the 198 patients in the cTACE group, although the difference was not significant. Most of the deaths were related to the tumor itself. Other causes included infections and complications of cirrhosis. Figure 1 compares survival between the DEB-TACE and cTACE groups, in which it was, respectively, 87.0% and 87.9% in one year, 35.1% and 32.9% in three years, and 20.5% and 18.1% in five years (*p* = 0.661).

When comparing the groups in terms of post-embolization complications, we found that the rate of adverse events was lower in the DEB-TACE group (7.1% vs. 17.8%), although the difference was not statistically significant (*p* = 0.052). In both groups, the most common complication was post-embolization syndrome.

## DISCUSSION

In the present study, survival did not differ significantly between the patients who underwent cTACE and those who underwent DEB-TACE. Similar results have been reported in some other studies and meta-analyses^(15,19-22)^. A large, multicenter randomized clinical trial conducted by Golfieri et al.^(15)^ (of the Precision Italia Study Group) showed that both techniques are equally effective and safe, with similar oneand two-year survival rates-86.2% and 56.8%, respectively, for DEB-TACE and 83.5% and 55.4%, respectively, for cTACE. Those are higher than the rates obtained in the present study, especially for the second year of follow-up. It is noteworthy that in the present study a complete radiological response was more common in the cTACE group, although that does not seem to have influenced survival. In contrast, two meta-analyses showed that survival is better after DEB-TACE than after cTACE^(23,24)^. Nonetheless, neither the American Association for the Study of Liver Diseases^(25)^ nor the European Association for the Study of the Liver^(26)^ suggest that one method is more effective than the other.

In our study sample, most of the patients underwent superselective catheterization, as recommended in the literature^(27)^, and catheterization as a rule was successful. Golfieri et al.^(28)^ reported that a complete response and tumor necrosis ≥ 90% were observed approximately twice as often when selective or superselective catheterization was used than when nonselective catheterization was used (*p* = 0.013 and *p* = 0.008, respectively). The complete response rate observed for cTACE in the present study was similar to that previously described at our center^(29)^, whereas that observed for DEB-TACE was lower, although similar results have been reported^(14)^. Although Golfieri et al.^(15)^ observed higher response rates for DEB-TACE, the difference was not statistically significant. The PRECISION V study^(14)^, which was a prospective, randomized phase II trial conducted in five countries, with a collective total of 212 patients, also showed no significant difference between cTACE and DEB-TACE in terms of the complete response rate (27% vs. 22%). One recent systematic review and meta-analysis, evaluating 34 studies involving a collective sample of 4,841 patients with HCC, in which the mean follow-up period ranged from 6 weeks to 18 months, showed no significant difference between the two TACE methods in terms of the complete or partial response rate^(30)^.

Despite not being the aim of this study, it seems interesting to reflect on the costs involved in performing these procedures. A study conducted in the United Kingdom showed an unadjusted mean cost difference of £3,770.30 for DEB-TACE in comparison with cTACE^(31)^. In that study, patients undergoing DEB-TACE required fewer treatment sessions, although there was a bias because those patients had significantly fewer target lesions. However, the reality in Brazil is different, given the high price charged by the companies that supply the microspheres for DEB-TACE and the fact that no cost-effectiveness studies of the procedure have been carried out in the country. In addition, the public health care system in Brazil only makes cTACE available to patients, excluding DEB-TACE because of the costs. However, given that we have demonstrated similar results, it seems reasonable to perform cTACE when and where DEB-TACE is unavailable.

As for the rate of adverse effects, there was no statistical difference between the two groups in the present study, although this finding may be controversial. In the PRECISION V study^(14)^, the proportion of patients with post-embolization syndrome was similar in both groups, although the increase in aminotransferases was less pronounced in the DEB-TACE group. The authors also showed that the difference in the left ventricular ejection fraction was smaller in the DEB-TACE group and that the frequency of gastrointestinal adverse events was lower in the cTACE group (45% vs. 61%). In the randomized trial conducted by Golfieri et al.^(15)^ (of the Precision Italia Study Group), the only observed advantage of DEB-TACE was a lower incidence of abdominal pain after the procedure. However, various systematic reviews have shown no difference in the adverse event rates^(16,23,24,30)^.

The importance of the present study lies in the fact that in Brazil^(32)^, as well as in Latin America at large^(33)^, TACE is the treatment most frequently offered to patients with HCC. In fact, for patients with HCC at an intermediate BCLC stage, TACE is the treatment of choice^(8)^. Despite the need for screening and surveillance of patients with cirrhosis in order to diagnose HCC earlier, that recommendation is not often followed in practice^(34)^. Therefore, in most cases, when HCC is diagnosed, it is no longer possible to offer curative treatment.

In the present study, as observed in other study conducted in Brazil^(32)^, the most common etiology of cirrhosis was infection with hepatitis C virus, whereas in the rest of the world, especially in Asia and Africa, the most common etiology is infection with hepatitis B virus^(35,36)^. In our patient sample, the age at diagnosis and distribution by sex are in agreement with data in the literature^(37,38)^. The AFP levels were low in our patients, which is in keeping with the findings of an epidemiological survey conducted in Brazil, in which most of the patients had an AFP level below 100 ng/mL^(32)^. As expected, the majority of patients in our study were categorized as Child-Pugh class A and had a MELD score < 15, given that decompensated cirrhosis is a contraindication for performing TACE^(26,39,40)^.

Our study has some limitations. First, the retrospective nature of the study limited its ability to identify temporal changes. In addition, the number of patients who underwent DEB-TACE was smaller than was that of the patients who underwent cTACE, which restricts the generalizability of the DEB-TACE results. Furthermore, some patients underwent CT to assess the response to the procedure. That, together with the fact that lipiodol can introduce artifacts and hinder the identification of enhancement on CT (potentially leading to a higher frequency of complete responses), represents another limitation.

In conclusion, our findings indicate that DEB-TACE has no significant advantages over cTACE. The two techniques appear to be comparable in terms of survival and the occurrence of adverse effects.

## References

[1] Petrick JL, Florio AA, Znaor A (2020). International trends in hepatocellular carcinoma incidence,
1978-2012. Int J Cancer.

[2] Bray F, Ferlay J, Soerjomataram I (2018). Global cancer statistics 2018: GLOBOCAN estimates of incidence
and mortality worldwide for 36 cancers in 185 countries. CA Cancer J Clin.

[3] McGlynn KA, Petrick JL, El-Serag HB (2021). Epidemiology of hepatocellular carcinoma. Hepatology.

[4] Massarweh NN, El-Serag HB. (2017). Epidemiology of hepatocellular carcinoma and intrahepatic
cholangiocarcinoma. Cancer Control.

[5] Sangiovanni A, Prati GM, Fasani P (2006). The natural history of compensated cirrhosis due to hepatitis C
virus: a 17-year cohort study of 214 patients. Hepatology.

[6] Chagas AL, Mattos AA, Carrilho FJ (2020). Brazilian Society of Hepatology updated recommendations for
diagnosis and treatment of hepatocellular carcinoma. Arq Gastroenterol.

[7] Llovet JM, Burroughs A, Bruix J. (2003). Hepatocellular carcinoma. Lancet.

[8] Reig M, Forner A, Rimola J (2022). BCLC strategy for prognosis prediction and treatment
recommendation: the 2022 update. J Hepatol.

[9] Yamada R, Sato M, Kawabata M (1983). Hepatic artery embolization in 120 patients with unresectable
hepatoma. Radiology.

[10] Brown DB, Geschwind JFH, Soulen MC (2006). Society of Interventional Radiology position statement on
chemoembolization of hepatic malignancies. J Vasc Interv Radiol.

[11] Chang Y, Jeong SW, Jang JY (2020). Recent updates of transarterial chemoembolization in
hepatocellular carcinoma. Int J Mol Sci.

[12] Lewis AL, Taylor RR, Hall B (2006). Pharmacokinetic and safety study of doxorubicin-eluting beads in
a porcine model of hepatic arterial embolization. J Vasc Interv Radiol.

[13] Melchiorre F, Patella F, Pescaroli L (2018). DEB-TACE: a standard review. Future Oncol.

[14] Lammer J, Malagari K, Vogl T (2010). Prospective randomized study of doxorubicin-eluting-bead
embolization in the treatment of hepatocellular carcinoma: results of the
PRECISION V study. Cardiovasc Intervent Radiol.

[15] Golfieri R, Giampalma E, Renzulli M (2014). Randomised controlled trial of doxorubicin-eluting beads vs
conventional chemoembolisation for hepatocellular carcinoma. Br J Cancer.

[16] Facciorusso A, Licinio R, Muscatiello N (2015). Transarterial chemoembolization: evidences from the literature
and applications in hepatocellular carcinoma patients. World J Hepatol.

[17] Bruix J, Sherman M, Practice Guidelines Committee, American Association for the Study of
Liver Diseases (2005). Management of hepatocellular carcinoma. Hepatology.

[18] Lencioni R, Llovet JM. (2010). Modified RECIST (mRECIST) assessment for hepatocellular
carcinoma. Semin Liver Dis.

[19] Karalli A, Teiler J, Haji M (2019). Comparison of lipiodol infusion and drug-eluting beads
transarterial chemoembolization of hepatocellular carcinoma in a real-life
setting. Scand J Gastroenterol.

[20] Facciorusso A, Di Maso M, Muscatiello N. (2016). Drug-eluting beads versus conventional chemoembolization for the
treatment of unresectable hepatocellular carcinoma: a
meta-analysis. Dig Liver Dis.

[21] Gao S, Yang Z, Zheng Z (2013). Doxorubicin-eluting bead versus conventional TACE for
unresectable hepatocellular carcinoma: a meta-analysis. Hepatogastroenterology.

[22] Savic LJ, Chen E, Nezami N (2022). Conventional vs. drug-eluting beads transarterial
chemoembolization for unresectable hepatocellular carcinoma-a propensity
score weighted comparison of efficacy and safety. Cancers (Basel).

[23] Chen P, Yuan P, Chen B (2017). Evaluation of drug-eluting beads versus conventional
transcatheter arterial chemoembolization in patients with unresectable
hepatocellular carcinoma: a systematic review and
meta-analysis. Clin Res Hepatol Gastroentrol.

[24] Han T, Yang X, Zhang Y (2019). The clinical safety and efficacy of conventional transcatheter
arterial chemoembolization and drug-eluting beads-transcatheter arterial
chemoembolization for unresectable hepatocellular carcinoma: a
meta-analysis. Biosci Trends.

[25] Marrero JA, Kulik LM, Sirlin CB (2018). Diagnosis, staging, and management of hepatocellular carcinoma:
2018 practice guidance by the American Association for the Study of Liver
Diseases. Hepatology.

[26] Forner A, Reig M, Varela M (2016). Diagnosis and treatment of hepatocellular carcinoma. Update
consensus document from the AEEH, SEOM, SERAM, SERVEI, and
SETH. Med Clin (Barc).

[27] Golfieri R, Cappelli A, Cucchetti A (2011). Efficacy of selective transarterial chemoembolization in inducing
tumor necrosis in small (<5 cm) hepatocellular carcinomas. Hepatology.

[28] Golfieri R, Renzulli M, Mosconi C (2013). Hepatocellular carcinoma responding to superselective
transarterial chemoembolization: an issue of nodule
dimension?. J Vasc Interv Radiol.

[29] Lionço LC, Mattos AA, Horbe AF (2017). Treatment of hepatocellular carcinoma using transarterial
chemoembolization: a real-life, single-centre study from Southern
Brazil. Eur J Gastroenterol Hepatol.

[30] Bzeizi KI, Arabi M, Jamshidi N (2021). Conventional transarterial chemoembolization versus drug-eluting
beads in patients with hepatocellular carcinoma: a systematic review and
meta-analysis. Cancers (Basel).

[31] Fateen W, Khan F, O’Neill RJ (2017). Healthcare costs of transarterial chemoembolization in the
treatment of hepatocellular carcinoma. J Hepatocell Carcinoma.

[32] Carrilho FJ, Kikuchi L, Branco F (2010). Clinical and epidemiological aspects of hepatocellular carcinoma
in Brazil. Clinics (Sao Paulo).

[33] Debes JD, Chan AJ, Balderramo D (2018). Hepatocellular carcinoma in South America: evaluation of risk
factors, demographics and therapy. Liver Int.

[34] Wolf E, Rich NE, Marrero JA (2021). Use of hepatocellular carcinoma surveillance in patients with
cirrhosis: a systematic review and meta-analysis. Hepatology.

[35] Maucort-Boulch D, de Martel C, Franceschi S (2018). Fraction and incidence of liver cancer attributable to hepatitis
B and C viruses worldwide. Int J Cancer.

[36] Yuen MF, Chen DS, Dusheiko GM (2018). Hepatitis B virus infection. Nat Rev Dis Primers.

[37] Akinyemiju T, Abera S, Ahmed M, Global Burden of Disease Liver Cancer Collaboration (2017). The burden of primary liver cancer and underlying etiologies from
1990 to 2015 at the global, regional, and national level: results from the
Global Burden of Disease Study 2015. JAMA Oncol.

[38] Fassio E, Díaz S, Santa C (2010). Etiology of hepatocellular carcinoma in Latin America: a
prospective, multicenter, international study. Ann Hepatol.

[39] European Association for the Study of the Liver (2018). EASL clinical practice guidelines: management of hepatocellular
carcinoma. J Hepatol.

[40] Kloeckner R, Galle PR, Bruix J. (2021). Local and regional therapies for hepatocellular
carcinoma. Hepatology.

